# A Clinical Decision Support System for Sleep Staging Tasks With Explanations From Artificial Intelligence: User-Centered Design and Evaluation Study

**DOI:** 10.2196/28659

**Published:** 2022-01-19

**Authors:** Jeonghwan Hwang, Taeheon Lee, Honggu Lee, Seonjeong Byun

**Affiliations:** 1 Looxid Labs Seoul Republic of Korea; 2 Department of Neuropsychiatry Uijeongbu St Mary's Hospital, College of Medicine The Catholic University of Korea Uijeongbu-si Republic of Korea

**Keywords:** sleep staging, clinical decision support, user-centered design, medical artificial intelligence

## Abstract

**Background:**

Despite the unprecedented performance of deep learning algorithms in clinical domains, full reviews of algorithmic predictions by human experts remain mandatory. Under these circumstances, artificial intelligence (AI) models are primarily designed as clinical decision support systems (CDSSs). However, from the perspective of clinical practitioners, the lack of clinical interpretability and user-centered interfaces hinders the adoption of these AI systems in practice.

**Objective:**

This study aims to develop an AI-based CDSS for assisting polysomnographic technicians in reviewing AI-predicted sleep staging results. This study proposed and evaluated a CDSS that provides clinically sound explanations for AI predictions in a user-centered manner.

**Methods:**

Our study is based on a user-centered design framework for developing explanations in a CDSS that identifies why explanations are needed, what information should be contained in explanations, and how explanations can be provided in the CDSS. We conducted user interviews, user observation sessions, and an iterative design process to identify three key aspects for designing explanations in the CDSS. After constructing the CDSS, the tool was evaluated to investigate how the CDSS explanations helped technicians. We measured the accuracy of sleep staging and interrater reliability with macro-F1 and Cohen κ scores to assess quantitative improvements after our tool was adopted. We assessed qualitative improvements through participant interviews that established how participants perceived and used the tool.

**Results:**

The user study revealed that technicians desire explanations that are relevant to key electroencephalogram (EEG) patterns for sleep staging when assessing the correctness of AI predictions. Here, technicians wanted explanations that could be used to evaluate whether the AI models properly locate and use these patterns during prediction. On the basis of this, information that is closely related to sleep EEG patterns was formulated for the AI models. In the iterative design phase, we developed a different visualization strategy for each pattern based on how technicians interpreted the EEG recordings with these patterns during their workflows. Our evaluation study on 9 polysomnographic technicians quantitatively and qualitatively investigated the helpfulness of the tool. For technicians with <5 years of work experience, their quantitative sleep staging performance improved significantly from 56.75 to 60.59 with a *P* value of .05. Qualitatively, participants reported that the information provided effectively supported them, and they could develop notable adoption strategies for the tool.

**Conclusions:**

Our findings indicate that formulating clinical explanations for automated predictions using the information in the AI with a user-centered design process is an effective strategy for developing a CDSS for sleep staging.

## Introduction

### Background

Polysomnography is a systematic process for collecting physiological parameters during sleep and is a diagnostic tool for evaluating various sleep disorders. Physiological recordings obtained from an electroencephalogram (EEG), electrooculogram (EOG), and electromyogram (EMG) were inspected by polysomnographic technicians to obtain important sleep parameters. Sleep staging is the process of identifying periodic changes in sleep stages. Typically, sleep stages are identified for every 30-second signal or epoch. On the basis of the American Academy of Sleep Medicine; wake status; 3 non–rapid eye movement (REM) stages, namely N1, N2, and N3; and REM stages were identified from polysomnographic recordings [1]. Sleep staging is an essential task in sleep medicine, as sleep patterns contain critical information for analyzing overnight polysomnography. To be specific, crucial sleep parameters, such as the distribution of sleep stages, were extracted from the sleep staging results. For example, the N1 stage, which is difficult to differentiate from the wake stages, is used to calculate the time to sleep onset and total sleep time parameters. The detection of REM stages affects the calculation of REM latency after sleep, which is another important sleep parameter. Furthermore, the physiological characteristics associated with each sleep stage have been investigated to diagnose several sleep disorders, such as obstructive sleep apnea, narcolepsy, and REM sleep behavior disorder [2,3]. However, in polysomnography, sleep staging is a time-consuming and costly process because every epoch in an overnight recording must be manually inspected. Several algorithms have been introduced to automate this time-consuming and costly task [4-6].

### Artificial Intelligence–Based Clinical Decision Support Systems for Sleep Staging

Advances in deep learning techniques have led to the development of clinical Artificial Intelligence (AI) systems with diagnostic performance comparable with that of human clinicians [4,7-9]. These models have been introduced to automate time-consuming diagnoses and annotation procedures in clinical fields. However, the full automation of diagnostic processes, where algorithmic counterparts completely replace human clinicians, is presently not available owing to several challenges: the reliability of model predictions [10], clinical soundness of model behaviors [11], and social consensus on the replacement [12]. Similarly, in sleep medicine, several studies have introduced AI algorithms to automate time-consuming sleep staging tasks, but manual reviews of the results after automated prediction remain mandatory [13,14]. Under these circumstances, systems to assist polysomnographic technicians during the review process are in demand. For example, prior work in human–AI interaction conceptualized a framework in which ambiguous portions in polysomnographic recordings are selectively prioritized for manual inspection [15].

Despite an increasing number of deep learning studies for sleep staging [4,5], implementing an adoptable clinical decision support system (CDSS) for clinical practice remains a challenging task. First, regarding clinical knowledge, most deep learning–based systems lack explainable factors, but clinical staff members require clinically sound systems [10,13,16]. Thus, the CDSS should provide users with the necessary explanations. Second, the user interface of the AI system should be practical in clinical environments, where the time and resources of clinicians are constrained [10,17]. Therefore, a tool design that promotes readability and accessibility of the AI model from the viewpoint of clinical practitioners is indispensable for integrating AI-based decision-making into the workflow of human technicians [10,18]. The development of such CDSSs is crucial because these tools could alleviate these time-consuming and costly clinical tasks. Furthermore, proper algorithmic assistance can enhance the performance of clinical practitioners [19].

### Study Objectives

In this study, we introduce an AI-based CDSS for assisting polysomnographic technicians when reviewing the AI-generated sleep staging results. Our objective is to correctly understand the information required from the CDSS and to develop the system in a user-centered manner. Through an extensive user study, we determined the features desired in a sleep staging AI system that could successfully support sleep technicians. We formulated the development process of a tool to assist clinical practitioners effectively.

## Methods

### Study Design

This study aimed to understand what information should be provided to assist sleep technicians in collaborating with AI-based CDSS and to implement this system practically using a user-centered approach. Recent studies for designing explanations in CDSS propose frameworks that identify three key components from the perspectives of users: *why* information from CDSS is desired for a task, *what* content should be included in the explanation, and *how* explanations should be presented to users [20,21].

To define why users need explanations from the CDSS, the context within which users request explanations must be understood first. This question relates to the needs of the users and the purpose of the explanations. The perspective of users should determine the explanatory objective concerning the information that should be provided. A possible set of information that can be considered from this phase includes explanations for the input data, explanations related to the domain knowledge used in the task, causal information on how the system generates an output, and how results change with changes in input data [21,22]. Finally, several design factors, such as the units and format used for explanations, are considered when determining how information should be provided.

**Figure 1:**
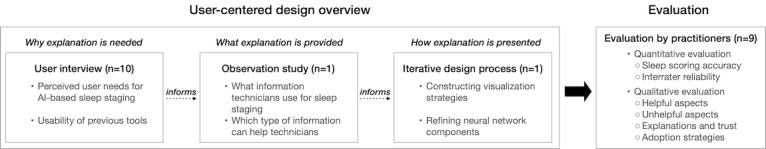
Overall development process. AI: artificial intelligence.

To design a CDSS within this framework, our development process included three phases: (1) interviews with polysomnographic technicians to identify why users might desire explanations from the CDSS when adopting AI-based sleep scoring systems, (2) user observations of how polysomnographic technicians score sleep stages from EEG recordings to determine the information that could help them, and (3) an iterative design process to construct a user-friendly CDSS interface that addresses the formulation of explanations in the system. After development, the polysomnographic technicians performed quantitative and qualitative evaluations of the system. In this section, we describe the objectives of each phase and explain how we conducted each phase (Figure 1).

### Participants

Polysomnographic technicians with expertise in sleep staging were recruited for this study. Only technicians with a national license for medical laboratory technologists who were eligible to conduct polysomnography scoring were considered. To recruit participants with expertise in sleep scoring, we restricted their participation to those with experience in polysomnography scoring. We recruited 10 technicians to participate in the user interviews during the first phase and subsequent evaluation studies. We set the number of participants to 10, following previous studies on CDSSs, in which the number of participants was between 6 and 12 [15,23]. Among the technicians, we aimed to recruit 1 technician who could deeply engage in the development process by participating in the user observation and iterative design processes, which required regular meetings. We recruited technicians from secondary and tertiary hospitals rather than primary hospitals. Participants were recruited through emails sent to the polysomnographic technician community.

We recruited participants and divided them into two groups, *novice* technicians with <5 years of experience and *senior* technicians with >5 years of experience, to evaluate whether there were any differences in the helpfulness of the CDSS based on the amount of experience. On the basis of the Rasmussen skill-, rule-, and knowledge-based behavior model [24], we assumed that senior technicians would score stages subconsciously compared with novice technicians who consciously process the EEG characteristics. Here, we expected that novice technicians would more extensively refer to the provided explanation than senior technicians because novice technicians may find it difficult to quickly locate important EEG patterns. Thus, it was thought meaningful to investigate how our explanations affected technicians based on their skills.

### Development Procedure

#### User Interview: Why Explanation Is Desired

We conducted user interviews with polysomnographic technicians to investigate why technicians would need explanations from the CDSS when AI-based support systems were adopted for sleep staging. During the interview, we first presented several questions regarding user needs during manual sleep staging and the perceptions of technicians regarding the utility of previous sleep staging AI tools. The technicians were asked whether they were using the automated sleep staging programs. Furthermore, the reasons for not adopting such automated sleep staging programs were investigated. Upon further investigation, we established the context in which explanations from AI were desired when reviewing automated sleep staging results. A user study was conducted using structured interviews with the sample questions listed in Textbox 1.

**Examples of interview questions in the user study.**

**Topic and question statement**

**User needs during manual sleep staging**

1. How much time do you spend on a sleep staging task when performing polysomnography?
2. For sleep staging tasks, on which features of electroencephalogram recordings do you mainly focus?
3. Do you feel any need for assistance during sleep staging?

**Utility of sleep staging artificial intelligence (AI) tools**

1. There are several AI programs that automate sleep staging tasks; are you adopting them in your workflow? If not, what are the problems associated with these programs?
2. In which processes do you need AI programs to assist your sleep staging tasks?
3. Assuming that there is an AI program that automates sleep staging tasks and sleep technicians only need to review its scorings, in which context are explanations desired for an efficient review process?

#### User Observation: What Information Should Be Contained in Explanations

A user observation study was performed to understand the sleep staging conventions of clinical practitioners. From the observed sleep staging conventions, we aimed to construct a list of EEG characteristics to which technicians refer. During this study, hour-long weekly meetings were held over a month in which a participating technician scored EEG epochs in a think-aloud protocol. The technician was requested to verbally express how the information in the EEG recordings during sleep scoring was processed. Afterward, the technician reviewed the scoring with detailed explanations of the reasons for scoring the epochs with the annotated stages. The objective of these observation sessions was to formulate what information could assist technicians in reviewing predictions from AI algorithms. The observations were made based on characteristic EEG patterns such as sleep spindles, k-complexes, and frequency waves listed in the sleep manual [1]. We investigated how the listed EEG characteristics were inspected in practice. Subsequently, we grouped the EEG features into typical explanations that our CDSS could provide.

#### Iterative Design Process: How Explanations Can Be Presented

We conducted an iterative design process with a technician to identify how explanations should be presented to CDSS users. For 2 months, we held weekly 2-hour meetings.

The Template-Guided Neural Networks for Robust and Interpretable Sleep Stage Identification from EEG Recordings (TRIER) was selected as the AI algorithm for generating explanations. It is a convolutional neural network architecture used to process single-channel EEG data for sleep staging, and was proposed to extract clinically meaningful EEG wave shapes. This study demonstrated the possibility that features in the convolutional filters could be related to important EEG characteristics such as sleep spindles and k-complexes, with a sleep staging performance comparable with human raters with macro-F1 scores of 0.7-0.8 on public sleep data sets. We considered three components in the TRIER, namely convolutional filters, saliency values, and intermediate activation, as sources of information for generating explanations. These three components have been widely used in interpreting neural network operations in previous machine learning studies [25-30]. Detailed technical descriptions of these components are provided in Multimedia Appendix 1 [1,29-32].

During the iterations, we aimed to investigate whether the information contained in the above components could provide the desired information obtained from the user observation study. In these sessions, the technician inspected the features obtained by the neural network components and expressed an opinion on whether they could provide sufficient explanation for the task. Information from the components was refined based on the feedback. Subsequently, we chose the exact component for generating explanations from the neural network components. However, because the information in neural networks is numerical, adequate visualization is required to enhance the user-friendliness of the explanations. Therefore, we iteratively collected feedback on the representation format of the explanations during the later sessions. The technician tested the prototype versions of the proposed tool and provided feedback in terms of their intuitiveness and helpfulness. Consequently, visualization strategies were constructed for the explanations and overall interfaces.

### Evaluation Study

#### Data Set Preparation

During the evaluation, technicians scored the sleep stages on sleep recordings from a public sleep EEG data set, the ISRUC-Sleep Dataset [33]. These data contain polysomnographic recordings obtained from 100 subjects with evidence of sleep disorders. This data set was collected from the Sleep Medicine Centre of the Hospital of Coimbra University. We adopted the public data set for sleep staging to calculate sleep staging performance based on the ground-truth labels provided in the data set. The characteristics of the data sets are summarized in Table 1.

The data were divided into a training set (80 participants), validation set (10 participants), and test set (10 participants). Only data samples from the training data set were used for training the deep learning models. We used the validation data set to select the model to be used for constructing the CDSS. The model with the best performance scores for the validation set was selected. The experimental results and corresponding findings were drawn exclusively from the test data set, which means that to avoid information leakage issues that may affect model accuracy, the data samples used for training the model were not used during the evaluation study.

To construct the data set for the evaluation study, we randomly extracted 15-minute EEG segments from the EEG recordings in the test data set. EEG segments with no changes in sleep stages were excluded from the selected segments. We evaluated the sleep scoring performance with 15-minute segments rather than whole-night polysomnography to evaluate the helpfulness of the tool effectively. Considering that technicians often skim through recordings and pay attention to EEG epochs with stage changes, the effectiveness of the system might not be revealed or hindered by the back-and-forth temporal relations between the sleep stages. This evaluation configuration was also adopted in a previous CDSS study for sleep staging [15]. In addition to the test set of 15-minute segments, we constructed a test data set composed of disconnected single epochs of EEG recordings to function as a stress test in which technicians must interpret the characteristics of an EEG epoch only from the EEG epoch without temporal relations derived from previous epochs. In these single-epoch test sets, because there are no previous or following epochs to provide information about the current epoch, the technicians can no longer rely on the scoring results from the previous epochs. The intention here was to clearly reveal the effectiveness of the explanations of the EEG characteristics.

In summary, our test data set consisted of two EEG settings: *a set of 15-minute* EEG *segments* and *a set of single-epoch* EEG *segments*. All the participants scored the same set of EEG recordings. A figure explaining our data setting is provided in Multimedia Appendix 1.

**Table 1:** Summary characteristics of the ISRUC-Sleep Dataset^a^ (N=100).

Characteristics		ISRUC-Sleep Dataset
**Gender, n (%)**		
	Male	55 (55)
	Female	45 (45)
Age (years), mean (SD)		51 (16)

^a^ISRUC-Sleep Dataset was scored based on American Association of Sleep Medicine Rules.

#### Experimental Setting

During the experiments, we compared sleep staging performance under 2 different settings. The first was sleep scoring using our CDSS against the baseline AI, where technicians scored stages with AI systems that included only AI predictions provided without any explanation. The second was sleep scoring using our CDSS versus a conventional setting, where technicians need to score each epoch without the predictions by AI. We configured the baseline AI and conventional settings to compare sleep staging settings for our CDSS.

To compare the sleep staging performance under different scoring settings, the technicians had to score each EEG epoch twice as follows: once each with our CDSS and the comparison setting. This was a fair comparison setting to evaluate the efficacy of the system because the characteristics of EEG segments affect sleep staging results significantly. Previous CDSS studies also employed this scoring setting to compare 2 different sleep staging support systems [15]. We divided the test data set into 2 groups and used the first to compare our CDSS with the baseline AI system. A different portion of the test data set was used to compare our CDSS with the conventional sleep staging setting. We randomly permuted the order of the EEG segments and the staging settings. Furthermore, there was a washout period before the second reading of the EEG to avoid the memorization effect.

#### Quantitative Evaluation

On the basis of the scoring results obtained from the experiments, we evaluated 2 important performance aspects for assessing sleep staging results. First, we considered the accuracy with which the technicians scored the sleep stages under different sleep staging settings. Studies on previous CDSSs have witnessed enhancements in diagnostic accuracy when using the developed CDSS [34-37]. Similarly, we investigated how explanations from our system affect the accuracy of sleep staging. To estimate the classification performance after reviewing the AI predictions, the *macro-F1 score*, which was adopted in previous studies for evaluating sleep staging performance, was used as a performance metric [4,6]. We calculated the metric using the sleep stage labels provided in the public data set as the ground-truth sleep stages. The macro-F1 scores were calculated for each 15-minute EEG segment and a portion of single-epoch EEG recordings.

Second, we evaluated whether interrater reliability was improved by adopting our CDSS. Interrater reliability between polysomnography technicians has been a critical issue in sleep staging because of the variability in interpreting polysomnography recordings among technicians [38]. Following previous work in sleep medicine, which demonstrated that an adequate information system could reduce interrater reliability [19], we investigated whether the information from our CDSS could enhance this property. With this objective, interrater reliability was measured using the *Cohen κ score* [39]. Given the sleep staging results for a 15-minute EEG segment, we calculated the Cohen κ score for every possible pairing of technicians under the same sleep staging setting.

In addition to the above metrics, we also evaluated whether participants could critically assess the accuracy of the model prediction in our system. We calculated the *correction rates of the predictions for incorrectly classified epochs*. Here, we measured the number of incorrectly predicted epochs revised by technicians and incorrectly predicted epochs revised to correct stages. We assumed that for incorrectly predicted epochs, the AI might generate erroneous explanations. Thus, it would be easier for participants to detect incorrectly predicted samples. To evaluate this aspect, we intentionally provided EEG epochs with incorrect AI predictions during the evaluation study.

#### Qualitative Evaluation

To investigate the extent to which the developed system supported polysomnographic technicians, we conducted semistructured postevaluation interviews. During the survey, we asked questions on a wide range of topics, such as the helpfulness of the information and how the participants adapted to the system. User trust in a system is an important aspect in designing AI-based CDSSs [16,40]. Thus, questions regarding user trust in the developed system were included in the postevaluation interviews. Questions regarding *how information from the system was used in the sleep staging process* were asked during the interviews to reveal notable adoption strategies. The sample interview questions are presented in Textbox 2.

**Examples of interview questions in the qualitative evaluation.**

**Topic and question statement**

**User experience of the tool**

1. Were the automated predictions and explanations provided in the clinical decision support systems helpful during the experiment? If not, which aspects were unhelpful?
2. How did you perceive the provided explanations when the automated predictions agreed or disagreed with your decisions? Did it affect your trust in the system?
3. Did the explanations correspond well to your perception of the important waveform patterns?

**Adoption strategy for the tool**

1. How did you use each explanation strategy during the experiment?
2. Was there any notable strategy for adopting the explanations rather than merely accepting the information in the explanations?

#### Statistical Analysis

As mentioned in the previous section, each EEG epoch was read twice under 2 different settings as follows: once with our CDSS and once with comparison methods, the AI system without explanations, or the conventional staging setting without AI predictions. A statistical comparison was conducted to investigate whether the sleep staging performance was enhanced by adopting our CDSS compared with the comparison settings. Rather than comparing the distribution of the scores, we performed a paired comparison analysis in which we compared 2 sleep scoring performances on the same EEG segments under 2 different score settings. As scoring results could be affected by the complexities and characteristics of particular EEG epochs, it is critical to control these variabilities when assessing the significance of each performance. Furthermore, to exclude variability arising from interrater differences and only consider enhancements in performance by adopting our CDSS, we exclusively performed within-subject analysis for the macro-F1 scores.

The Wilcoxon signed-rank test, a nonparametric statistical test for a set of matched samples [41], was used to estimate the significance of the improvements by adopting the proposed test. For every participant, the data pairs were configured as follows: the macro-F1 and Cohen κ scores from the baseline or usual sleep staging setting (*μ_1_ κ_1_*) and the classification results when adopting our CDSS (*μ_2_ κ_2_*). For macro-F1 scores, for performance pairs from the same technician, there could be a clustering effect. Thus, we used the Wilcoxon signed-rank test for clustered data, which can account for clustering effects [42-44]. This test aimed to reveal whether performance was significantly enhanced by pairwise comparison when controlling for the variance arising from the interrater characteristics and the differences in EEG epochs. The significance of the results is reported by in terms of *P* values. We set the significance threshold at .05. All statistical and significance tests were performed using Python 3.6. We calculated the *P* values, sample sizes (n), *z* statistics, and effect sizes (*r*) using the Wilcoxon signed-rank test [45].

### Ethical Considerations

The study was approved by the institutional review board of the Uijeongbu St Mary’s Hospital (IRB number UC20ZADI0137), which waived the requirement for informed consent owing to the nature of the study. All EEG recordings used in this study were acquired from public data sets. All data were anonymized to ensure confidentiality.

## Results

### Participants Characteristics

In total, 10 polysomnographic technicians were recruited from 3 different affiliations, 2 tertiary hospitals, and 1 secondary hospital. A total of 10% (1/10) of the technicians participated in the user interview, user observation sessions, and an iterative design process. We refer to this participant as technician A throughout the *Results* section. The other 90% (9/10) of the technicians participated in user interviews and evaluation studies. Among the 10 participants, 40% (4/10) were novice technicians with <5 years of experience. A total of 60% (6/10) were senior technicians with >5 years of experience. Technician A, who participated in the tool design process, was excluded from the evaluation study to avoid bias in favor of our CDSS. The participant characteristics are summarized in Table 2.

**Table 2:** Participant characteristics.

Demographics		Novice technicians (n=4)	Senior technicians (n=6)
Experience (years), mean (SD)		1.75 (1.3)	12.5 (4.7)
**Affiliations, n (%)**			
	Secondary hospital	2 (50)	1 (17)
	Tertiary hospital	2 (50)	4 (83)

### User Interview: Why Explanation Is Desired

#### Reasons Technicians Did Not Use Automated Scoring Tools

In total, 20% (2/10) of the participants had no experience of using automatic sleep scoring programs; the other participants preferred not to refer to the automated sleep staging results during sleep staging. The technicians answered that even when predictions were automatically recommended by the software, they removed the automated predictions and scored all the epochs themselves.

In addition to the inaccuracies of algorithms, 50% (5/10) of the participants pointed out that *a lack of explanation* was the main barrier to adopting AI. One technician stated that, “The tools I have experienced do not provide any explanations for predictions, and I need to score every epoch all by myself again when reviewing the predictions.” Participants further called for the *clinical soundness of their explanations*. Another technician answered as follows:

There certainly exist clinical features to focus on for sleep staging. Even if automatic programs provided some sort of explanation, we need to check whether clinically appropriate EEG features, such as sleep spindles or amplitudes of alpha waves, are used in the algorithms.

These assertions reflect important considerations regarding explanations and the clinical soundness of algorithm procedures when designing a CDSS [13,16,46].

#### The Context in Which Explanations Will Be Used

As stated in the subsection above, technicians requested that AI programs should provide clinically sound explanations for predictions, as reviewing the correctness of AI predictions without this information is no different from the manual annotation of sleep stages from scratch. Participants were requested to suggest desirable AI adoption scenarios during the interviews.

In total, 80% (8/10) of the technicians wanted *clinically sound explanations of the predictions*. This is relevant to correct EEG patterns that are important for scoring sleep stages, where users can easily assess the correctness of the reasoning from the AI model based on the conventional manuals for the clinical task:

Some automatic programs seem to use procedures that differ from the widely adopted conventions shared among sleep technicians. I think information from AI should adhere to the procedures that we were trained with to make it easier for us to assess the rationale for the explanations.

Another technician said as follows:

When reviewing the AI predictions, I need grounds that convince me. As we are trained to stage based on standard manuals, explanations from AI should be closely related to these processes.

This point is especially critical in the clinical domain, where predefined sets of rules exist [10].

To summarize the trend of the interview answers, the technicians wanted explanations to validate the correctness of the AI predictions based on their clinical knowledge of sleep staging.

### User Observation: What Information Should Be Contained in Explanations

By observing technician A for 1 month, we obtained an understanding of how technicians interpret EEG signals during sleep staging. Using the clinical context proposed in the manual [1], we categorized EEG patterns based on how the technician processed the information in the EEG recordings. On the basis of how they processed each EEG feature, we created a list of explanation types that can be provided in the CDSS. The candidate explanation-type categories are listed in Textbox 3.

**Explanation type to be provided in the clinical decision support systems.**

**Explanation type 1: occurrence of signals**

For some patterns in electroencephalogram recordings, their presence is a clear indicator of certain sleep stages. For example, the occurrence of *sleep spindles* and *k-complexes* is strongly correlated to non–rapid eye movement (REM) 2 stages. In general, technicians search the entire signal to find these patterns. Therefore, proper detection of these patterns is sufficient information for polysomnographic technicians.

**Explanation type 2: ratio of signals**

Technician A claimed that estimating the ratio of *delta waves* in an epoch is the most critical part in identifying the non-REM 3 stages since the scoring manuals recommend annotating the epoch as stage non-REM 3 when delta waves account for more than 20% of the signals [1]. The participant mentioned that technicians usually count the number of delta waves manually to correctly identify the non-REM 3 stages in sleep recordings.

**Explanation type 3: changes in signals**

Alpha waves are prevalently observed during the wake and non-REM 1 stages. However, the participant mentioned that changes in the amplitudes of *alpha waves* are important criteria for distinguishing non-REM 1 stages from the wake stages. According to the manual [1], the alpha waves in the non-REM 1 stages normally exhibit smaller amplitudes compared with the wake stages. Technician A mentioned that perceiving the overall changes in alpha waves is the primary task in detecting boundaries between the wake and non-REM 1 stages.

### Iterative Design Process: How Explanations Can Be Presented

#### Refinements of Model Components

**Figure 2:**
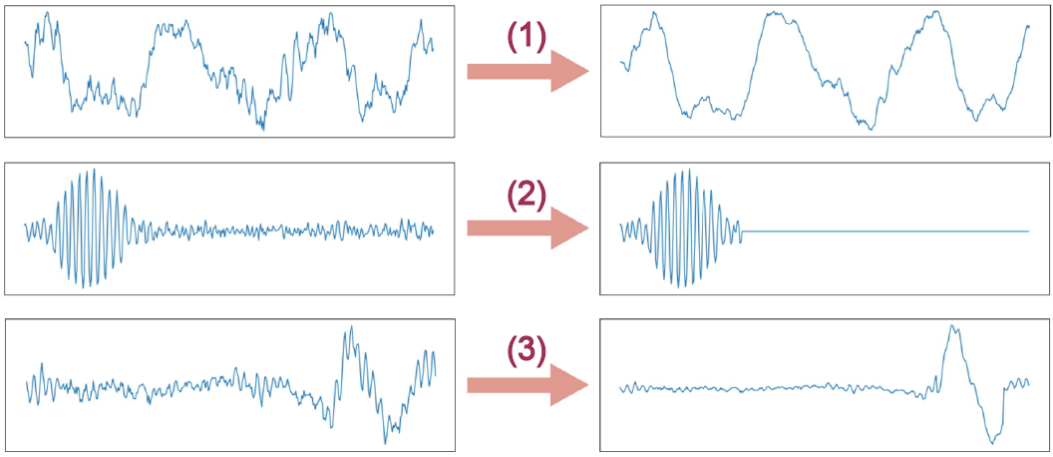
The filter refinement process is as follows: (1) delta waves were low-pass filtered, (2) regions outside the sleep spindle were zeroed-out, and (3) only the regions corresponding to k-complex features were selected and low-pass filtered afterward.

In the first iteration session, convolutional filters obtained from TRIER [28] were shown to technician A. The participant expressed the concern that although the convolutional filters contained morphologically significant shapes, undesirable features (high-frequency noises or low-frequency fluctuations) were also intermingled in the filter. The participant requested a refinement of the convolutional filters to improve the quality of the features. For example, in formulating filters that correspond to slow waves, the participant wanted to remove high-frequency components because delta waves have frequency components <4 Hz. The filter refinement process is illustrated in Figure 2. Consequently, the convolutional filters contain features that correspond to the following EEG patterns: alpha waves, theta waves, delta waves, sawtooth waves, vertex sharp waves, sleep spindles, and k-complexes. After refinement, the filters are depicted in Multimedia Appendix 1.

#### Selecting Information Source for Making Explanations

Owing to the previous refinement process, components in the convolutional filter are clinically meaningful, and the corresponding features in the neural networks can be interpreted accordingly. For example, for a filter that was designated for k-complex–related features, the activation values generated from the filter were used to locate k-complexes in the data. Similarly, filters analogous to alpha waves can generate information related to alpha wave changes in the data.

Therefore, we selected convolutional filters and activation values as basic elements to generate explanations of the model predictions. In addition to the 2 components, a saliency map [29], or the gradient values of the input points, was also adopted to mark significant regions in making a prediction. This information indicates which regions in the data were important from the AI perspective. The neural network components used for generating explanations are summarized in Textbox 4.

**List of information sources for generating information for the clinical decision support systems.**

**Component 1: convolutional filters and their activation values**

Convolutional filters represent the clinical electroencephalogram patterns on which the model is based. Information regarding each clinical feature can be obtained from the activation values acquired from the filters.

**Component 2: saliency values calculated from neural networks**

Important regions, which significantly contributed to model predictions, can be inspected from the saliency values. Users can view the data from the perspective of the artificial intelligence model with saliency values.

#### Visualization Strategies

Visualization strategies for each clinical feature were devised to provide information in an easily adopted form for sleep staging. Initially, plots of activation vectors without any processing were provided to the participating technician. In this case, the technician failed to use any of the information in the activation values. They emphasized that information should be compatible with the scoring procedure of the technician: “I cannot make use of the information. I want information to be provided in a form that can easily fit with my procedure.” This argument is closely linked to the critical issues in designing AI assistant tools: information from the system should be easily integrated into tasks of users [47,48].

From this standpoint, we constructed different visualization strategies for each explanation type because conventions observed during the user observation study constituted the representative logical procedures for processing information in EEG recordings (Textbox 5).

**Figure 3:**
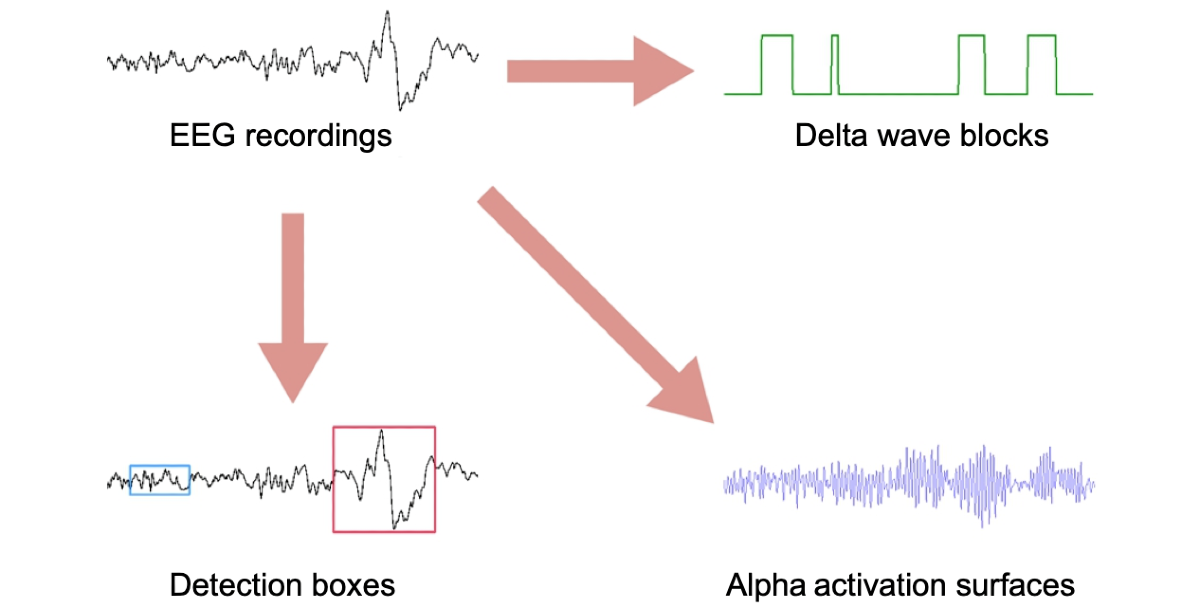
Visualization strategies for each interpretation pattern. Information in electroencephalogram (EEG) recordings is visualized differently for each interpretation convention introduced in Textbox 5.

Figure 3 shows an in-tool visualization of the strategies. Through visualization, explanations from AI can be conveyed to users with their proper clinical contexts. Technician A attested that such explanations with enhanced readability could be easily adopted in the sleep staging process.

**Four visualization strategies developed in this study. The first three strategies correspond to the interpreting conventions observed during the user observation study.**

**Strategy 1: detection boxes**

Technician A claimed that the patterns, the presence of which alone indicates a sleep stage, should be more easily identified from the recordings. After that, it would be sufficient for the technicians to check whether the artificial intelligence (AI) model correctly located these patterns in the electroencephalogram (EEG) recordings. Therefore, we outlined detection boxes in regions that were detected to include the desired EEG patterns. Detection algorithms were implemented based on the amplitudes of the activation values calculated from the convolutional filters with the desired pattern.

**Strategy 2: delta wave blocks**

As polysomnographic technicians rely on the number of delta waves in the recordings, it is important to make the distribution of delta waves more visually intuitive. For these cases, technician A wanted to perceive each peak in delta waves as a single entity. We digitized the activation values from the convolutional filters of delta waves such that regions with activation values higher than a set threshold were encoded as 1 or otherwise as 0. Visualizing the encoded digits from the activation vectors, technician A perceived the information as blocks of slow waves and counted the number of blocks in the figure.

**Strategy 3: alpha activation surfaces**

For detecting changes of alpha waves on the boundary of the wake and non-REM 1 stages, the activation values generated directly from the convolution between the alpha wave filters and the input recordings were used. In this case, the participant requested fluctuations to be easily perceivable in the interface. During the iterations, technician A acknowledged that overall fluctuations of the activation values matched well with the perception of the changes. In such a setting, it was felt that the activation values amplify the changes in amplitudes. The technician asserted that these values are perceived as a surface area, thus making it more intuitive to sense overall changes in the signal.

**Strategy 4: saliency highlights**

The participant claimed that saliency values could be helpful for technicians as they could view the recordings from the AI perspective. In particular, the technician wanted to identify the EEG regions with high saliency values. Therefore, we highlighted the EEG recording segments with high saliency values.

#### Constructing the System Outline

In the original version of the system, we empower users to explore EEG recordings interactively with a filter selection box with which users could choose the desired EEG patterns and analyze signals based on the selected features. However, technician A observed that the system with an exploratory filter selection process might degrade its usability, as it disrupts the workflows of the technician:

Usually scoring of an epoch takes place in a short time, typically between five to ten seconds and even down to one second for easy cases. The selection process can be a bottleneck during scoring, thus other technicians are more likely to skip filter selection and score EEG epochs on their own.

This indicates that for clinical tasks where large numbers of data points are annotated in a relatively short time, the accessibility of desired features could be more important than interactivity. Therefore, instead of interacting with multiple features, we implemented an information system to be directly accessible.

**Figure 4:**
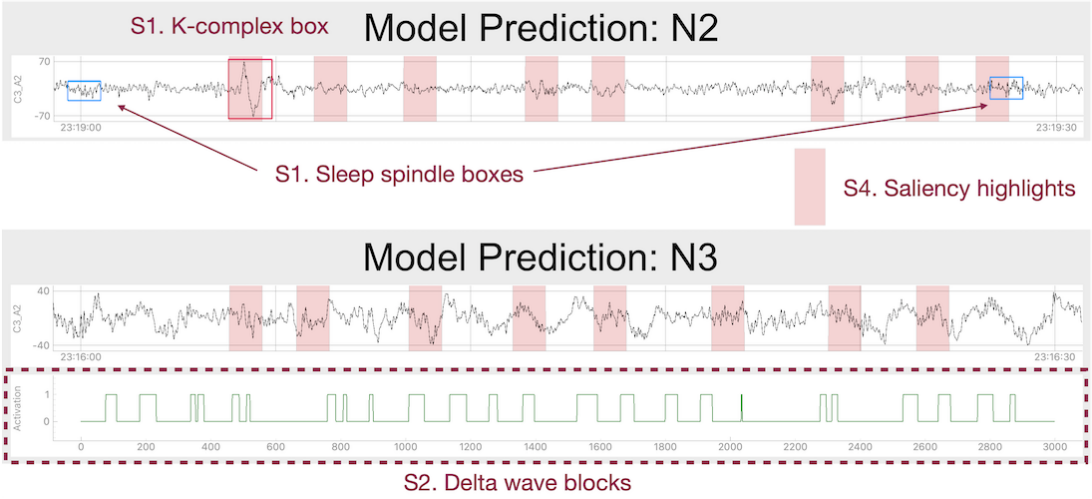
Visualization strategies for the system. In the electroencephalogram (EEG), the recordings predicted as N2, k-complex, and sleep spindles are detected and visualized as red and blue boxes. In EEG recordings predicted as N3, detected delta waves are visualized as green blocks. Regions with high saliency values are highlighted in pink on the EEG recordings. Strategy is abbreviated as S.

Specifically, rather than providing multiple sets of available information, we chose to show only the information corresponding to the predicted sleep stage for the epoch (Figure 4). For example, only the detection boxes of sleep spindles and k-complexes were provided for the epochs that were predicted as N2 stages. In this version, technician A acknowledged that the usability of information is enhanced compared with previous versions where multiple sets of information are provided, which results in too much information on a single screen and poor readability. Furthermore, the visualizations could explain the model predictions because the model provides only information relevant to its predictions. In Table 3, we list specific information provided for each stage.

**Figure 5:**
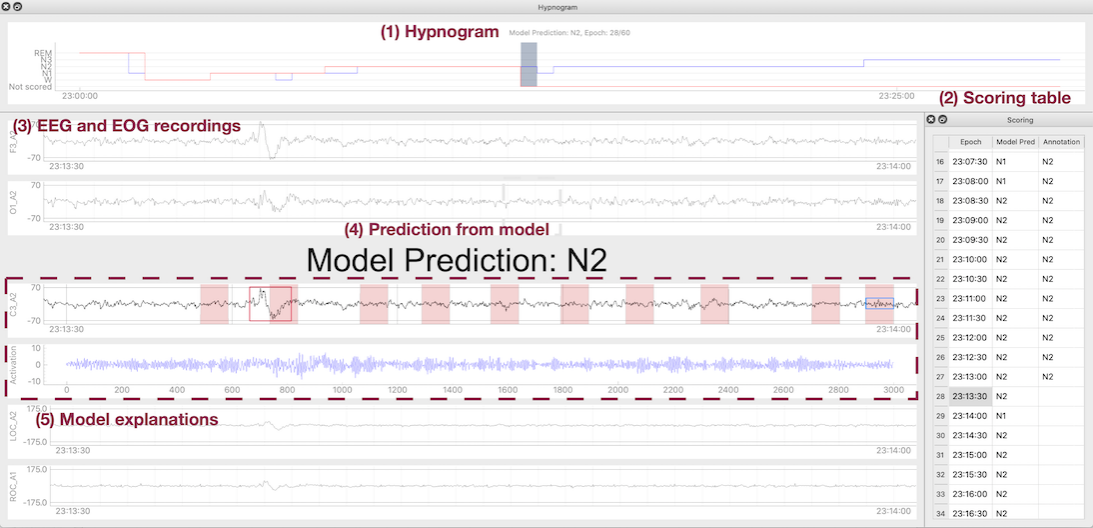
The following is the overall interface of the system: (1) hypnogram; (2) scoring table lists the time sequence of model predictions and user annotations; (3) physiological recordings of the data set are visualized in the main panel; (4) predictions; and (5) explanations from artificial intelligence (AI) are in the middle of the interface. EEG: electroencephalogram; EOG: electrooculogram.

Similar to other tools for assisting sleep staging [49], our system provides basic information from EEG recordings (Figure 5). It displays the hypnogram, a graph that visualizes changes in sleep stages over time, on top of its interface. Hypnograms for annotated stages from users as well as predictions from AI are provided so that users can monitor their editing process. A table that contains time information and annotated sleep stages is located on the right panel of the interface. The EEG and EOG recordings of an epoch are depicted in the main interface. In addition to the basic components, our CDSS provides the following information: AI-generated predictions and explanations from the AI model around the target EEG channel. Video recording provided in Multimedia Appendix 2 demonstrates the overview of the CDSS and how users interact with it.

**Table 3:** Information provided for each sleep stage.

Stage	Detection boxes	Delta wave blocks	Alpha activation surfaces	Saliency highlights
Wake			✓	✓
N1^a^			✓	✓
N2^a^	✓		✓	✓
N3^a^		✓		✓
REM^b^	✓		✓	✓

^a^N1-3: non–rapid eye movement stages 1-3.

^b^REM: rapid eye movement.

### Quantitative Evaluation

#### Accuracy

**Figure 6:**
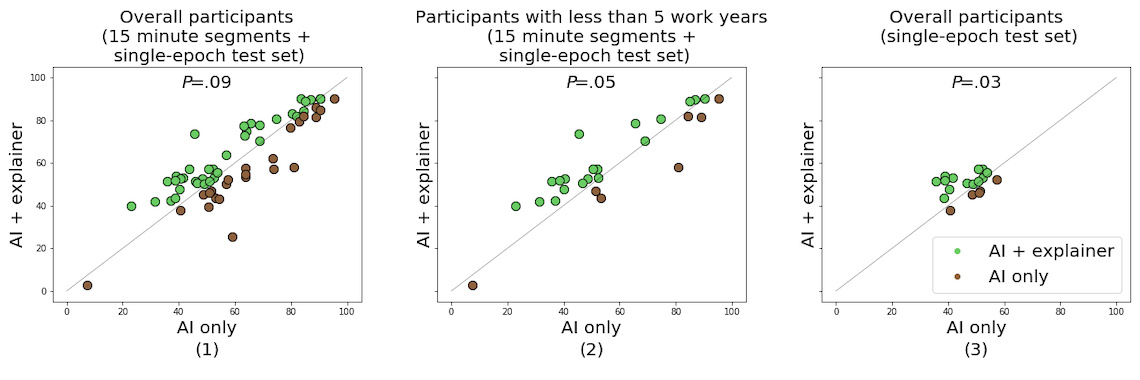
The improvements of the macro-F1 scores in various settings. The results measured as follows from (1) all participants and all test sets; (2) participants who have <5 years of work experience and all test sets; and (3) all participants and single-epoch test sets are provided. AI: artificial intelligence.

Figure 6 illustrates macro-F1 scores. Each point in the scatter plots corresponds to the performance pair measured using the comparison method (AI only, *μ_1_*) and our method (AI+explainer, *μ_2_*) on the same test set.

For the overall data set, which consisted of 15-minute EEG segments and single-epoch test set, there were no significant differences between baseline AI and our CDSS for results from all participants (*µ*_1_=60.22; *µ*_2_=61.31; *P*=.09; n=26; *z*=1.63; number of clusters=9)*.* However, a performance improvement can be observed when we restricted this data set to participants with <5 years of work experience (*µ*_1_=56.75; *µ*_2_=60.59; *P*=.05; n=26; *z*=1.63; number of clusters=4). For a single-epoch test set, in which the utility of the methods could be more accurately determined, we also observed improvements in accuracy (*µ*_1_=46.55; *µ*_2_=50.28; *P*=.03; n=18; *z*=1.94; number of clusters=9).

For the overall data set, compared with the conventional staging setting where predictions from the AI were not provided (*µ_1_*), the macro-F1 scores were significantly improved when the technicians adopted our method (*µ*_1_=43.23; *µ*_2_=68.04; *P*=.004; n=17; *z*=2.64; number of clusters=9). Similarly, the macro-F1 scores improved for novice technicians when we compared our CDSS with a conventional sleep staging setting (*µ*_1_=39.52; *µ*_2_=70.58; *P*=.05; n=6; *z*=1.67; number of clusters=3).

It should be noted that these results cannot be directly compared with sleep staging performance in other studies where performance was evaluated for whole-night sleep staging results. In our setting, the performance was measured from short segments of the EEG recordings. Here, sleep staging performances could be reported to be lower than the whole night sleep staging results in previous works, as the macro-F1 scores of the sleep staging results could be significantly affected by a few incorrect predictions.

#### Correction Rates for Incorrect Predictions From the AI

For the erroneous predictions generated by the AI, statistics regarding the ratio of correctly revised epochs did not show significant differences between the baseline AI and our method. Among the 392 EEG epochs in the test data, 30.8% (121/392) were incorrectly predicted epochs from our network. Of the 30.8% (121/392) of the epochs, technicians detected 28.5% (112/392) of the incorrect predictions made with our CDSS, whereas 28.5% (112/392) were detected in the baseline AI. There were no significant differences in the detection rates of incorrectly predicted epochs (*P*=.39; n=9; *z*=0.28;*r*=0.11). Furthermore, among these incorrectly predicted epochs from AI detected by technicians, there were no significant differences in the ratio of correct revisions where technicians identified the correct stages for incorrect predictions (*µ*_1_=15.68%; *µ*_2_=16.42%; *P*=.76; n=9; *z*=−0.70; *r*=0.28). Similarly, for technicians with <5 years of experience, we did not observe improvements in the detection rates of incorrectly predicted epochs (*µ*_1_=27.19%; *µ*_2_=30.52%; *P*=.86; n=9; *z*=−1.10; *r*=−0.60) and the ratio of correct revisions (*µ*_1_=12.90%; *µ*_2_=16.67%; *P*=.97; n=9; *z*=−1.83; *r*=−1.0)*.*

#### Interrater Reliability

**Figure 7:**
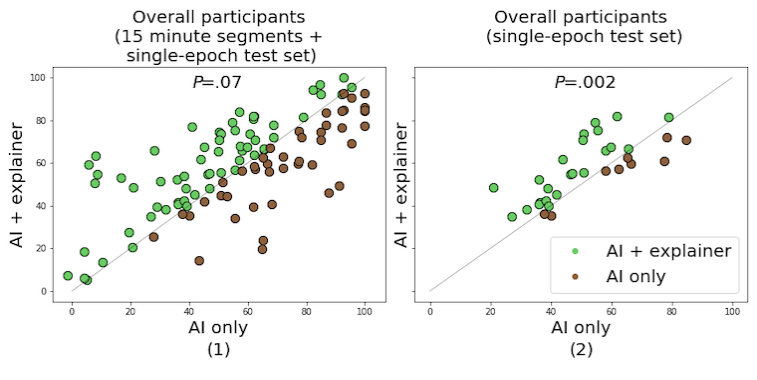
The improvements of interrater reliability in various settings. The results measured from the following: (1) all participants and all test sets and (2) all participants and single-epoch test sets are provided. AI: artificial intelligence.

Scatter plots of the Cohen κ scores calculated for the baseline (κ_1_) and our method (κ_2_) are shown in Figure 7. As with the macro-F1 scores, improvements in reliability for all cases were observed (κ_1_=57.02; κ_2_=59.54; *P*=.07; n=212; *z*=1.49; *r*=0.17). However, more significant improvements were observed for the single-epoch test set (κ_1_=51.28; κ_2_=57.21; *P*=.002; n=64; *z*=2.80; *r*=0.57). According to the criteria for interpreting the Cohen κ score [50], we obtained moderate agreement between technicians for both the proposed CDSS and baseline AI settings. Compared with usual sleep staging settings, where predictions from AI are not provided, interrater reliability also improved (κ_1_=35.06; κ_2_=77.48; *P*<.001).

### Qualitative Evaluation

In this section, a qualitative evaluation of the tool is described. The adoption strategies developed by the participants and the perceived usability of the system are discussed.

#### Helpful Aspects

In total, 78% (7/9) of the participants responded that our system helped to review AI predictions. They reported that they referred to information from the CDSS when inspecting AI predictions. Several aspects of the utility of the tools were confirmed.

#### Reducing the Workload Required for Pattern Recognition

One of the most important utilities mentioned during the interviews was that our tool reduced the workload required to inspect EEG epochs. Analyzing EEG epochs is similar to visual searching tasks, where technicians must identify specific patterns in a visual environment [51]. Participants attested that information visualized by saliency highlights and detection boxes drew their attention to important regions [52]. Helped by the information provided by the detection boxes, participants were easily able to identify important regions for examination. On the basis of this information, they assessed whether the patterns were correctly detected by the algorithm. Similarly, for delta waves, participants replied that they only needed to count the number of delta wave blocks, and they did not need to check delta waves one by one from the EEG recordings.

#### Providing Quantitative Visual Reference

Interviewees stated that sleep staging tasks heavily depend on the subjective criteria of each technician. Perceiving the attenuation in alpha waves on the boundary of the wake and N1 stages is one of the most representative cases in which sleep staging is affected by subjective perception. In total, 55% (5/9) of the participants used the information from the alpha activation surfaces as a reference when they were not confident whether changes in the alpha wave were significant. Even the participant who was not satisfied with the system answered that this information was helpful for similar reasons.

#### Unhelpful Aspects

Two senior participants answered that they did not find the system helpful. They claimed that the specificity of the information from the system was below the desired level:

I am quite strict in detecting sleep EEG patterns like delta waves. However, from my point of view, too many regions were annotated as significant points. Thus, for many cases, I did not refer to the provided information.

This point emphasizes that for clinical tasks where decision-making may differ between individuals, personal differences among users should be considered to improve the usability of a tool. In our domain, for example, user interfaces that control the sensitivity and specificity of the pattern detection algorithm can be provided.

Another technician did not refer to the system during the experiments because it was inconvenient to consider information other than the EEG recordings:

Due to time constraints, I am used to scoring stages speedily compared to other technicians. Thus, in some sense, I tend to rush during sleep staging sessions, and would rather not care about information in the system.

Interviews from the participants reveal that the tight time constraints in clinical environments are another challenge to be considered when designing a clinical support tool because changing the workflow of medical staff is a complicated task, which requires not only reliable performance but also usability in the workflows [46].

#### Explanations and Trust in the System

In this section, we summarize how the explanations of our systems affect user trust during the experiments.

#### Explanations in Agreed Epochs

For epochs in which the predictions of the participants agreed with those of the AI, the technicians expressed trust in the predictions. In this case, the participants expressed that, as annotated regions from the system matched the important regions determined by the users, they were confidently able to continue to the next stages.

#### Explanations in Epochs With Disagreement

For the epochs where the predictions differed between the AI and users and were consequently modified by the technicians, the participants felt that the explanations clarified why AI predicted the epochs differently. In these cases, one technician argued:

Without explanations, I might jump to a conclusion that the accuracy of the AI is not at a desirable level. However, after being exposed to the explanations, there were some convincing factors in the AI-generated predictions, and I tried to re-investigate the recordings based on the AI explanations to find out whether my reasoning on predictions was strong enough to modify the AI prediction.

Even the technician, who did not think that the tool was helpful, reported:

At first, I totally disagreed with the predictions from the AI. Throughout the experiments, however, I found out that AI algorithms were reasonable on some level.

In summary, even though user trust could be severely affected when the AI predictions were inconsistent with those of the users, the explanations provided in our CDSS improved the trustworthiness of the system. In particular, the explanations helped users find reasonable aspects of AI predictions.

#### Notable Adoption Strategies

We obtained various sets of answers such as “I first focused on saliency highlights and then inspected signals based on the detection boxes” or “I used the alpha activation surfaces in detecting sleep arousal.” Among these answers, some notable strategies were identified. We discuss these strategies and their implications for human–AI collaboration in clinical domains.

#### Rediscovery of Unnoticed Features

Classifying REM stages solely from EEG recordings is deemed an impossible task, and sleep technicians prefer to rely on chin EMG and EOG recordings for REM stages [1]. Thus, most participants had difficulty evaluating sleep epochs that were predicted as REM. However, several participants found that they could distinguish REM from the N1 stages with our method:

In general, I used to disregard sawtooth waves because REM has more distinct landmarks in EOG. However, the AI model correctly captured the sawtooth waves (patterns that occur in REM stages) and convinced me that the given epochs are from REM. Without such information, I might incorrectly score the stages.

These use cases demonstrate that our tool successfully conveyed important but easily dismissed features of the data. We believe that the above insight illustrates an important aspect of human–AI collaboration because alternative but significant viewpoints from the AI system successfully convinced the users during decision-making, which resulted in a performance enhancement.

#### Attention Allocation

In the adoption of a clinical AI system, to allocate their attention efficiently to weak portions of the algorithms, it is important for users to properly understand the strengths and weaknesses of AI. This scenario is termed the attention allocation [18]. During the experiments, several technicians developed strategies related to attention allocation. One participant found that AI is vulnerable to misidentifying sweat artifacts as delta waves. This participant strategically allocated more attention to annotated regions in epochs that were predicted as N3 stages and inspected whether the annotated regions corresponded to delta waves or sweat artifacts. With this strategy, this participant effectively distinguished the N3 stages from epochs contaminated by sweat artifacts.

In this adoption pattern, participants constructed strategies to successfully collaborate with AI [48]. Specifically, users evaluated the convincing and unconvincing contributions of AI, thus efficiently allocating their attention during the adoption.

## Discussion

### Principal Findings

To our knowledge, this work is the first to construct an interpretable AI system using deep learning with a user-centered approach to develop a CDSS for sleep staging. Recent studies continuously demonstrate that deep learning algorithms can achieve comparable performance compared with human experts [7-9]. However, previous studies have found that human practitioners require information beyond the delivery of accurate predictions [18]. To achieve this, we focused on constructing a CDSS that provides information compatible with the diagnostic patterns of human raters and helps technicians easily integrate the CDSS into their sleep staging procedures. Through user observation and an iterative design process, we obtained the desired characteristics for the explanations provided in the CDSS for sleep staging. First, clinical practitioners wanted explanations to help them validate AI predictions. Here, technicians wanted explanations that adhered to their clinical knowledge. Second, we categorized the type of information based on our observations of how technicians interpret the characteristics of each EEG. Finally, during the iterative design process, we confirmed that information contained in neural network components can be used to generate explanations for sleep staging results. The design components were updated iteratively based on the feedback of the technician.

When evaluating the improvements in the sleep staging performance of all participants, we did not observe significant improvements when the *P* value was approximately .17. However, we believe that our quantitative evaluation contains meaningful results. First, when assessing the improvements for novice participants, we observed that the macro-F1 scores improved by 6.7% with a *P* value of .02. Considering that novice technicians may rely more on supportive information than expert technicians, this result implies that our tool could be effectively used to augment the sleep scoring capacities of novice technicians with acceptable sleep-relevant explanations. Second, when assessing the improvements in a single-epoch sleep scoring setting, which is similar to a stress-test configuration, we observed significant improvements in the macro-F1 scores and interrater reliability. Notable results in this stress test setting could indicate that our explanations to an extent helped technicians interpret the signal characteristics of each EEG epoch. Third, the results of the qualitative evaluation implied that the CDSS supports sleep staging by reducing the workload required for pattern recognition and providing quantitative visual references. These findings show that the developed system successfully and appropriately complemented the assessments of the technician by suggesting the desired information. Our tool obtained such utility for two reasons: (1) clinically sound features were correctly addressed and (2) information visualization was designed to be acceptable in conventional workflows of the sleep staging process.

We identified further issues that should be considered when designing a CDSS. During the experiments, 20% (2/10) of the technicians indicated that our system was not adoptable for workflow in sleep staging. In particular, 10% (1/10) of the technicians expressed a lack of trust in the AI system. In general, the avoidance of algorithmic results is an important challenge to be addressed when adopting an automatic system [53]. However, these challenges can be interpreted based on skill levels of the technician. For example, based on the Rasmussen skill-, rule-, and knowledge-based behavior model [24], senior technicians may score sleep stages without consciously processing EEG information. Therefore, additional explanations from the CDSS can distract such technicians. In contrast, novice technicians may require additional cognitive processing of information in the recordings. Therefore, explanations from the CDSS could be helpful as guiding information during processing and lead to significant enhancements in their performance when a CDSS is adopted.

In addition, over reliance on computer systems is another challenge to be considered when adopting decision support tools [11,54]. When adopting AI systems, there are cases where users tend to accept predictions from systems without any personal judgment on whether the information is correct. In our evaluation, the correction rates for erroneous predictions did not improve. This means that even though explanations from our system successfully operated as convincing components for model predictions, they failed to reveal ambiguous predictions. These results have implications for further development (eg, explanations for uncertainties in predictions can be provided by the model to inform users about ambiguous components in the data [15]). The confidence of the predictions can be algorithmically estimated by the models as additional information [55]. Such features can be integrated into a single framework to enhance safety in human–AI interaction systems.

### Comparison With Previous Work

Previous studies on sleep staging have confirmed that suggestions for proper computational features can enhance sleep staging performance. An experimental study demonstrated that interrater reliability among technicians can be significantly improved by computer-derived suggestions [19]. Taking inspiration from that study, our work proposes an approach to provide clinically meaningful information from deep learning models. Our results are consistent with those of a previous study, as the interrater reliability in our system improved significantly. However, our study differs from previous works in several respects. Although previous tools for sleep staging have already provided sleep-relevant information to users [56,57], these algorithms require a large amount of parameter tuning to fit each data set [58]. In this sense, these works used a manually curated algorithm rather than augmenting the AI system to provide information. Furthermore, our work addresses the utility and readability of the system during the development of the tool, whereas previous studies preferentially focused on the calculation of sleep-relevant features in EEGs.

In the domain of human–AI interaction, several deep learning models have been exploited as information sources to assist medical staff with appropriate knowledge. In these works, the usability of clinical AI was mainly addressed from the perspective of human users [18]. A previous study surveyed how and what information should be provided for the analysis of radiographic images [59]. This work stressed that information systems should be designed based on the user needs of clinical practitioners. Another study introduced a novel medical image retrieval system that leverages embedding vectors in a neural network to retrieve similar medical images [47]. These bodies of work demonstrated that model interpretations should be formulated in the context of clinical knowledge, as users require medical explanations during adoption. Similarly, our work extensively investigates the desirable characteristics of sleep staging AI and proposes how these features can be provided in a CDSS.

For sleep staging, an earlier work proposed an AI framework that prioritizes ambiguous epochs in EEG recordings with explanations in cases of uncertainty [15]. However, this study proposes a conceptual framework rather than a practical implementation of the system. In this work, CDSS was simulated in a Wizard of Oz experiment, where human researchers manually generated the explanations in the system to address the ambiguous epochs in EEG recordings. In contrast, our work proposed a practical methodology for constructing meaningful information on sleep stages to assist clinical practitioners.

### Limitations and Future Directions

The limitations that require consideration remain in our study. First, we conducted user observations and iterative design sessions with only 1 technician. Although manuals for sleep staging support most of the feedback of the technician, specific requirements defined by different users are necessary for user-centered design research. Moreover, during the experiments, participants reviewed the EEG recordings provided from a public EEG data set. As EEG recordings are highly heterogeneous across data sets and recording environments, the utility of the system could be more accurately evaluated if the neural network model was trained on data sets recorded in real-world settings.

Our work is further limited as we only considered EEG recordings for sleep scoring. Assuming real-world sleep scoring is performed with polysomnographic recordings, which include EEG, EOG, EMG, and ECG signals, not considering other recordings may have affected the scoring results. For example, eye movement patterns are crucial factors in identifying the REM stages. As we have only provided information for EEG recordings, we could not offer explanations regarding eye movements. However, we believe that our overall design approach can be applied similarly in future studies to explain the output of other physiological sensors, such as EOG and EMG. These future studies could construct a more comprehensible CDSS for sleep scoring. In addition, evaluation of the CDSS system with whole-night polysomnography will provide more generalizable performance results that can be connected to the results of real-world polysomnography.

The overall sample size may not be sufficient for comparison, considering that there are high interrater disagreements on the sleep staging results depending on individual characteristics. Even though we observed some notable improvements with the small sample size, a further evaluation study with more technicians is desirable. Furthermore, the representativeness of participants should be mentioned. Technicians from secondary and tertiary hospitals participated in the evaluation study, and technicians in primary hospitals were not considered. Technicians in primary hospitals may exhibit different tendencies toward the adoption of automatic sleep scoring tools. Thus, our study did not address this population. However, considering that technicians in primary hospitals tend to have relatively short experience in polysomnography, we believe that these results from novice technicians can be generalized to polysomnographic technicians in primary care.

An AI system that provides explanations for predictions was compared with conventional models that do not provide explanations. In this setting, there was a risk that the participants were aware that the experimental objective was to construct and evaluate the effectiveness of the explanations. However, considering that explainable AI systems for medical domains have not been widely developed, many previous CDSS studies conducted experiments in a similar manner to our work [15]. Nevertheless, the omission of blinding conditions is a limitation of our experimental setting.

Although our work qualitatively evaluates how users perceive the CDSS, future work is required to quantitatively assess the usability of the tool. For example, the NASA-Task Load Index [60] could be used in a prospective study to compare the required workload for each sleep scoring tool. Other aspects, such as time spent scoring sleep stages, could be estimated in a more controlled experimental setting. We believe that future studies will provide more insights into the usability of CDSS.

### Conclusions

Our findings indicate that formulating clinical explanations for automated predictions using information from an AI system that incorporates a user-centered design process is an effective strategy for developing a CDSS for sleep staging. The proposed CDSS has great potential to be integrated into the real-world clinical workflow in a sleep laboratory based on the extent to which performance was improved and is highly useful in sleep staging.

## Appendix

Multimedia Appendix 1 List of sleep-relevant electroencephalogram (EEG) patterns, refined convolutional filters, constructing data sets with EEG segments, and convolutional neural network components.

Multimedia Appendix 2 Demo video of the clinical decision support system.

## Abbreviations

AI: artificial intelligence
CDSS: clinical decision support system
EEG: electroencephalogram
EMG: electromyogram
EOG: electrooculogram
REM: rapid eye movement
TRIER: The Template-Guided Neural Networks for Robust and Interpretable Sleep Stage Identification from EEG Recordings
