# Supplementary material for: A Clinical Decision Support System for Sleep Staging Tasks With Explanations From Artificial Intelligence: User-Centered Design and Evaluation Study
Source: J Med Internet Res. 2022 Jan 19;24(1):e28659. doi: 10.2196/28659 (PMC8811694; doi:10.2196/28659)
Supplement: Multimedia Appendix 1 [file jmir_v24i1e28659_app1.docx]

## Multimedia Appendix 1

### List of EEG Patterns

All participants wrote down a list of important EEG patterns that are recommended in sleep staging manuals. We briefly provide a description of each pattern and its related stages in the table below. List in the Table contains most commonly answered patterns from the participants. We further describe with which sleep stages each pattern is correlated.

| Pattern | Key Characteristics | Stages |
| --- | --- | --- |
| alpha wave | high frequency waves (8-13 Hz) with relatively small amplitudes | Wake, N1, REM |
| theta wave | oscillatory patterns (4-7 Hz) with bigger amplitudes compared to alpha waves | N1, REM |
| delta wave | slow waves (0.5-4 Hz) with large amplitudes (75 $\mu$V) | N3 |
| sleep spindles | quick burst of oscillation (11-15 Hz) with small amplitudes | N2 |
| k-complexes | large transients in waveform consisted of negative and positive peaks | N2 |
| sawtooth waves | train of sharp waves that resemble sawtooth | REM |

### Overall Filters

Filters exploited in the experiment are shown in the figure. Filters are grouped by their patterns—filters after refinement. Participants' annotations are also written for each filter. For example, participants annotated that filters in the first and second columns correspond to features of alpha waves occurring during Wake. Based on these annotations, activation vectors were visualized and analyzed during implementation.


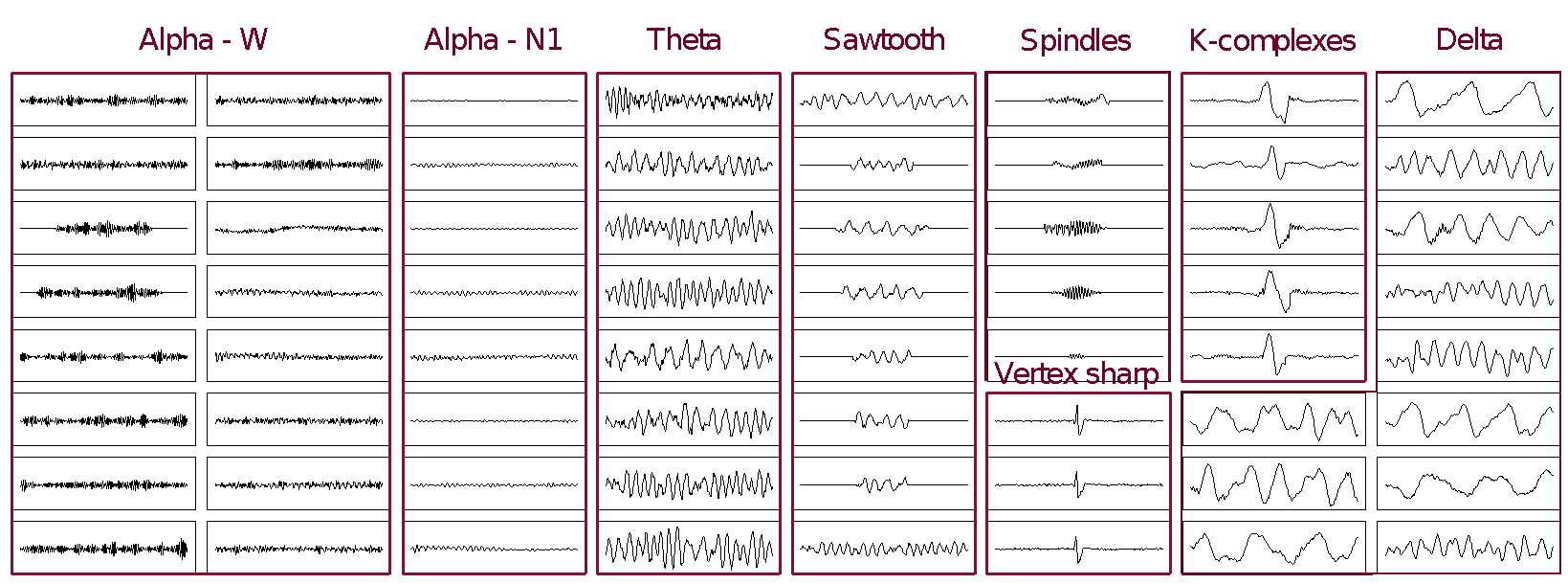


### Constructing Datasets with EEG Segments

From the test dataset, we extracted 15-minute segments and single-epoch test segments. Details on extraction are shown in the figure below. For selecting each 15-minute segment, we randomly selected 15-minute segments in the entire recording and gathered them into a candidate set of segments. Afterward, we selected the segments that contain changes in sleep stages from the candidate set. For each single-epoch test segment, 30-second epochs of the recording were randomly selected. Selected epochs were then concatenated in random order to make segments. Both 15-minute and single epoch segments were shuffled in the dataset. In the experiment, we have proposed two comparison settings: Baseline AI versus the Proposed CDSS and Manual Scoring versus the Proposed CDSS. We divide the whole test set into two groups, and each group was assigned exclusively for each condition. In each condition, segments are read two times under two different sleep scoring configurations according to the comparison setting. We set the washout period to avoid memorization of previously scored segments.


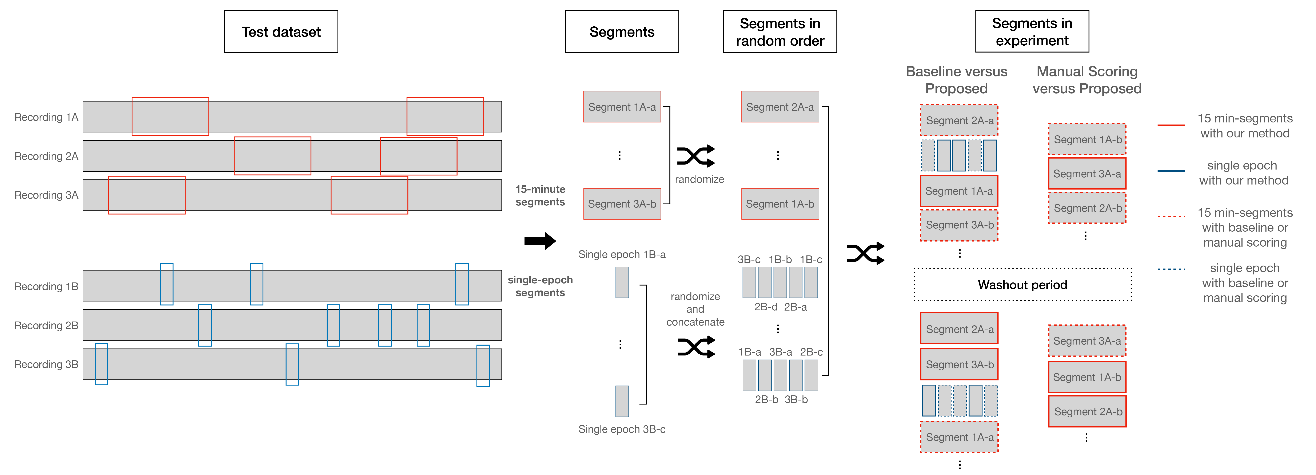


### Convolutional Neural Network Components

We give detailed explanations on neural network components used in the proposed method.

In a classification task, classifiers are given the input data and produce the predicted class as an output. However, putting the original data directly into a classifier leads to poor classification performance. Input data should be processed into a condensed form of information, feature, with which the classifier better works out. To find better features, various feature extraction techniques have been researched in the machine learning field. Nevertheless, the feature extractor is manually engineered since it shows different performances when the characteristics of input data vary. Therefore, feature extractors have mainly been designed by experts of the corresponding domain. In addition, feature extractors are independent of the classifier, which indicates the feature extracting process should start from scratch when the architecture of the classifier varies. Therefore, the feature extraction process requires a large amount of time and cost, with its performance still inconsistent with performance from different datasets and classifiers.

On the contrary, convolutional neural network (CNN) include feature extractor and classifier in themselves. That is, not only classifiers but also the feature extractors are trained automatically. The main objective of CNN models is, hence, to build the feature extractor that automatically recognizes the patterns from the input data and utilizes these patterns to classify the input data. For this goal, convolutional layers hierarchically process the information from the data [1, 2]. To be specific, in lower convolutional layers (layers that are close to an input layer), simple patterns (such as vertical or horizontal lines from the image) are recognized and learned. Afterward, complex patterns that are composed of the patterns from the lower layers are learned in higher convolutional layers. The fact that we can find these important patterns automatically from CNN models is the main reason we adopted them.

In sleep EEG recordings, there are important EEG patterns whose existence in the recording is directly related to deciding the sleep stage. As sleep technicians also look for these patterns in the sleep recordings and annotate them [3], they will produce better performance if our work can visualize various pattern-related components of CNN models into explanations. In this perspective, we introduce three components: convolutional filters, activation values, and saliency map. We explain how these components are related to explanations and why they are adopted in our CDSS.

#### Convolutional filter

Convolutional filters are the main result of training feature extractor. They filter out the patterns from the input data using convolution function. Therefore, we can say convolutional filters of each convolutional layer act as a receptive field of the fed data. A significant number of trained filters convey the information about recognized patterns to the next convolutional layer, if any. Passing through the convolutional layers, patterns that convolutional filters recognize get more complex. For example, in face recognition task [2], filters from the lower layer might recognize the edge of each shape such as vertical line, horizontal line or diagonal line. Then in the intermediate layer, combined patterns such as eyes, nose or mouth will be recognized. At the last convolutional layer, patterns resembling the human face will be recognized.

Convolutional filters generally show which EEG patterns are recognized by the CNN model. In this perspective, convolutional filters were shown to and refined by sleep technicians to help them understand the AI model is based on the clinically meaningful EEG feature and to show on which filters activation values are based.

#### Activation values

Activation values, features, are calculated from the convolutional filter and the data. If values of some areas are high, it indicates that the data contains the patterns of the convolutional filter. For example, for the audio classification task, activation values calculated from the filters that include frequency information are interpreted as pitch-related information [4]. Similarly, as filters in our CDSS represent clinically meaningful patterns, high activation values indicate some information relevant to clinical features contained in the corresponding convolutional filter.

Adopting this component, we expected the sleep technicians to be provided the brief information on how much convolutional filters match the input data. Utilizing this information, they can locate significant EEG patterns from the recording input more precisely.

#### Saliency map

Seeing a trained CNN model as one function composed of a feature extractor and a classifier, the input to it is EEG recording data and the output is prediction on the stage of that data. In this perspective, we can calculate the derivative of the model's output prediction with respect to the input data. This gradient value is called saliency map. Intuitively, when the gradient values are large, the prediction is greatly affected by changes in input data. Accordingly, when some region of input data is high in saliency values, we can say this region is significant for models’ prediction. In previous work, saliency map has been utilized as a basic component of AI models in highlighting the areas of the given image that discriminates a certain class [5].

When a model’s predicted class and saliency map for the prediction are provided to sleep technicians, they can see the model prediction in the AI model’s perspective. We believe it is important that sleep technicians know the fact that the AI model follows the rules they have acquired. We adopted saliency map as the explanation component to let human sleep technicians understand the AI model’s rationale for predicting sleep stages.

## Reference

[1] Yosinski, Jason, et al. "Understanding neural networks through deep visualization." *arXiv preprint arXiv:1506.06579* (2015).

[2] Albawi, Saad, Tareq Abed Mohammed, and Saad Al-Zawi. "Understanding of a convolutional neural network." *2017 International Conference on Engineering and Technology (ICET)*. Ieee, 2017.

[3] Berry, Richard B., et al. "The AASM manual for the scoring of sleep and associated events." *Rules, Terminology and Technical Specifications, Darien, Illinois, American Academy of Sleep Medicine* 176 (2012): 2012.

[4] Pons, Jordi, and Xavier Serra. "Randomly weighted cnns for (music) audio classification." *ICASSP 2019-2019 IEEE international conference on acoustics, speech and signal processing (ICASSP)*. IEEE, 2019.

[5] Simonyan, Karen, Andrea Vedaldi, and Andrew Zisserman. "Deep inside convolutional networks: Visualising image classification models and saliency maps." *arXiv preprint arXiv:1312.6034* (2013).
